# N. Y. State Academy of Veterinary Science and Comparative Pathology

**Published:** 1885-10

**Authors:** 


### Proceedings Veterinary Medical Societies.

### N. Y. STATE ACADEMY OF VETERINARY SCIENCE AND COMPARATIVE PATHOLOGY.

The first meeting of this season (after summer vacation) was held at Association Hall, West New Brighton, Staten Island, on Sept. 11, 1885.

The afternoon session was opened by the President, H. E. Earl, M.D.V.S., Chas. A. Meyer, D.V.S., Recording Secretary. The following members answered roll call: Drs. Hamil, Peters, Plageman, Hepburn, Reilly, Middleton, Juhl, Lindsay, Uibel, Sutcliff, Freeman, Schmidt and Rhors. Regrets were received from Drs. Conklin, McLaughlin, Dancer, Robertson, Laidlaw and Gill. Regrets were also received from friends of the Academy—Hon. George William Curtis, Erastus Brooks and Ernest A. Congdon, Recording Secretary of the Natural Science Association.

The preliminary business was to finish up the work of the June meeting by the appointment of a committee, with full power, to investigate the pathology of glanders. The instructions to this committee were to use all reasonable means in their power for this end, and to work both *separately* and *jointly* in their researches for the benefit of the Academy, the profession, and the public at large, and to report from time to time the results of their work.

Dr. Blumenthal, of Connecticut, gave his views of the difficulty of diagnosing glanders in its incipient stage.

The following were elected members of the Academy: A. E. Bannister, M.R.C.V.S., Alberta, Canada; Richard Ebbitt, M.R.C.V.S., Albany, N. Y.; Philip Brady, S.P.C.A., Clifton, S. I.

Dr. Meyer read a paper on "Mammitis in the Cow."

Dr. Middleton wished to know what was the best preventitive for this particular disease. The opinion of the Academy was that previous to parturition in cows overdue, milking the animal would be the best and safest preventitive.

Dr. Hamill asked that as mammitis was a form of erysipelas, and that the mineral terms were successful in the case, why would they not be useful in the horse, as it was a common affection or disease of the extremities of the horse during winter in New York.

As this was an original and useful subject, the Academy agreed to leave it open for further discussion at a future meeting.

Dr. Plageman then read the following paper on "Colic; its causes, complication, results and treatment":

I shall describe three forms of colic, viz.: spasmodic, flatulent and stercoral, all of which are more or less due to a spasm or contraction of the muscular coats of the intestines, and chiefly the colon; hence its name.

Spasmodic colic arises from various causes, generally from an animal partaking too freely of cold water or food when heated from physical exertion; from indisposition, constipation, the existence of worms, tumors, calculi, or intersusception, and from the application of cold or wet to the abdomen or legs; displacement of the cæcum, or large colon, or strangulation of the small colon, or laceration of any portion of the intestines are of frequent occurrence.

Flatulent colic is chiefly induced by a fermentation of food caused by special articles of diet, such as green clover or corn, green vegetables or fruit, if raw. Indian meal, or any element that is likely to become acid, or is already so, which produces an accumulation of gas in the intestines, and is frequently voided per anus and occasionally through the mouth.

Crib biting and wind sucking are predisposing causes, and sharp or uneven molars will cause the animal to swallow the food unmasticated, and producing tympanites.

The symptoms of spasmodic and flatulent colic are nearly similar, though there are distinguishing features. In the former, the patient is often more violent, pawing considerably, kicking at the abdomen, lying and rolling, and suddenly rising; pulse full and quick during the spasms, and breathing accelerated; sweats profusely; looks around at the side, and then there are intervals of perfect quiet.

In flatulent colic there is more or less distension of the abdomen and the pain more persistent; pulse apt to be feeble and quick, with increased respiration—the latter due to inordinate pressure on the diaphragm; hangs the head; the extremities are often cold, with cold sweats of the body; the two latter being very unfavorable signs.

In aggravated cases, where the distension is intense, the patient, if not speedily relieved, will die, either of asphyxia or rupture.

Stercoral colic, or impactment, known also as constipation, is caused by an accumulation of fæces, and is occasionally accompanied with tympanites. In all cases of constipation the patient will assume a position as if about to urinate, standing with all four legs stretched from under the body, and the veterinarian is often informed by the attendant that stoppage of water is the ailment. This peculiar position, I claim, is due to pressure by the obstruction or distension on the bladder.

I am of opinion that constipation is as likely to produce colic, as colic is to produce constipation.

Colicky pains frequently accompany special diseases or operations; for instance, purpura, tetanus, influenza, laminitis, suppuration cellulites, the operation of castration. Then there is colic induced by poisons or other irritants.

Therefore, I maintain that, according to the cause, so should the patient be treated. Yet it is a common thing for any one who has ever handled a a horse, whether he has groomed or driven him, or in the slightest degree had anything to do with a horse, to have some favorite remedy for colic. Many such nostrums, we are all aware, positively destroy life; then the violent exercise that the poor animal is subjected to by running or trotting him when suffering acute pain, with a view to expel gas or facillitate the peristaltic action, frequently kills the helpless creature.

Then when you ask such a person, would you have recourse to similar treatment if similarly affected? He would reply: No! I am not a horse. Certainly not; but I should judge he is of the species not far off.

In Stercoral colic the animal will lie for a considerable length of time, or stand, seldom rolls; pulse and respiration scarcely disturbed, except when the spasms occur; patient refuses to drink; mouth and tongue hot and dry; absence of intestinal murmur on auscultation; frequently a disposition to back up against some foreign object; resists enema, which are uncolored when ejected; sometimes sits on the haunches like a dog, which is not to be viewed as a favorable sign.

Should there be intersusception or rupture, calculi or tumor in the two former, death will invariably terminate the case, whilst in the latter two, the attacks may be more frequent, and the patient may survive many before he eventually succumbs.

Whilst I have briefly alluded to the different causes and symptoms of colic and its complications, I will say a few words on the distinguishing symptoms of enteritis, which is frequently associated with, and in which colic or constipation may terminate.

In enteritis the pain is continuous, visible mucous membrane injected, frequent sighing, increased respiration, cold sweats, cold extremities, mouth clammy, pulse wiry and quick, and feeble or strong, according to age, condition, temperament, etc.

Anxious expression and a dejected appearance in the last stages, pupils dilated, pulse imperceptible and a death tremor is quite apparent. In nearly all cases of colic relief is obtained by friction; there is an absence of fever, and quiet during a relaxation of the spasm.

I will not attempt to name the different nostrums that are chiefly resorted to, for they are probably familiar to you all, but I will mention that for over sixteen years I have used nothing else but the anodyne known as hydrate of chloral.

I believe it was first generally used about the year 1869; but it was discovered by Liebig nearly forty years ago. The doses I administered are from ʒii. to ʒiv. either in ball or in aquous solution. I know of many practitioners who give considerable more, and criticise my small doses, but I would advise any one who has never tried the small dose to do so. Camphor is another anodyne, which calms nervous irritation, and is diaphoretic. I occasionally combine the two, as they easily unite and form a syrup. The doses of the latter in combination is from ʒi. to ʒii. When there is much flatulency, ammonia is an admirable antacid to combine with chloral. I seldom have to administer a second dose, and if the patient is constipated I never administer a cathartic until the spasms are relieved; then I give an alortic cathartic, of from ʒv. to ʒviiii. of aloes, according to the size, age, breed and condition of the patient.

Looseness, or apparent diarrhœa, is often a percursor of constipation. Then I attribute it to indigestion. Even here a cathartic is necessary, but the dose should be modified.

Constipation may continue for as many as six or seven days, and during that time pain is present to a greater or lesser degree; then the remedy to relax spasm or relieve pain should be altered. For instance, I having given chloral in the first instance, might repeat the dose. but do not venture on a third. I change to ext. belladonna, opium or morphine, and do not follow up too closely. I would rather depend on hot fomentations to the abdomen and frequent enemas of plain warm water, and though the enemas do not reach further than the rectum, it acts as a sedative, and certainly does no harm.

After the first cathartic has been given, it is necessary to wait for at least thirty hours before a second dose should be given. I find that a combined purgative acts better in some cases. Then I give raw linseed oil or castor oil, and if the constipation is obstinate, and there is no symptoms of enteritis, 1 add a few drops of croton oil, say ten to twenty gtt. The patient seldom drinks and as liquids are inserted I drench with warm water.

Puncturing the intestines in tympanitis is a perfectly safe operation, and I have frequently given relief by so operating; but it is useless to puncture when the animal is in a dying condition, though I have punctured when it was impossible to administer the medecine, and afforded such instant relief that with other external and internal treatment following, I have saved the patient.

Tobacco smoke enemas I have often resorted to in many obstinate cases of constipation with beneficial results, acting as a narcotic, relaxing spasm, then slightly stimulating the mucous coat, it having the power of reaching where fluids cannot.

A member asked for information in regard to pleuro-pneumonia contagion in the vicinity of New York, especially Suffolk and Richmond Counties, where it was especially reported to exist.

Dr. Lindsay claimed that in his district he did not know of any cases, and was happy to state so for the benefit of his patrons.

Dr. Earl reported that it was in existence to a very limited extent in Richmond County, and could be easily stamped out by the proper precautions and inoculation.

Dr. Meyer claimed that in the city of New York it was in existence, and that it was likely to remain so under the present "sanitary laws."

J. F. Reilly, M.D., then read a paper on the physiological character of the alimentary tract.

