# Errata

**Published:** 1886-01

**Authors:** 


ERRATA.—In Dr. Plageman's paper on Colic in the October number of the journal, page 365, read Indigestion instead of Indisposition, Aliment instead of Element, Suppurative instead of Suppuration.

